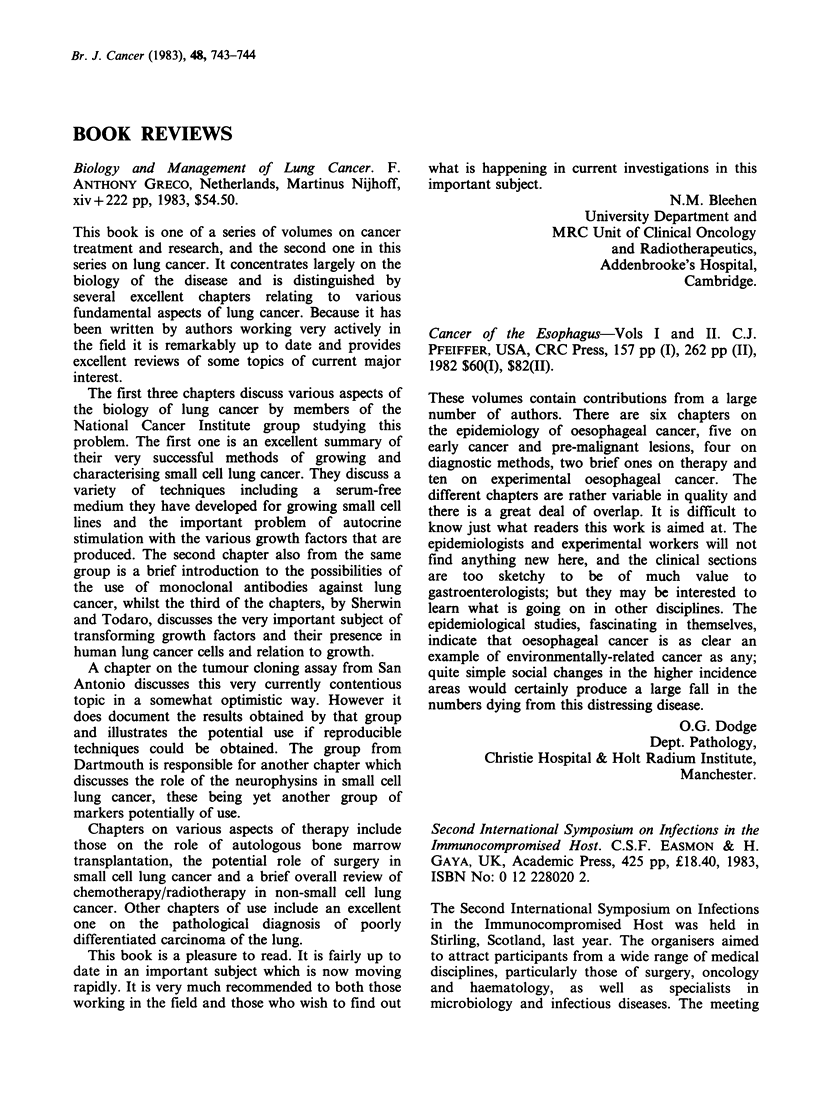# Biology and Management of Lung Cancer

**Published:** 1983-11

**Authors:** N.M. Bleehen


*Br. J. Cancer* (1983), **48**, 743–744

### BOOK REVIEWS

*Biology and Management of Lung Cancer*. F. ANTHONY GRECO, Netherlands, Martinus Nijhoff, xiv+222 pp, 1983, $54.50.

This book is one of a series of volumes on cancer treatment and research, and the second one in this series on lung cancer. It concentrates largely on the biology of the disease and is distinguished by several excellent chapters relating to various fundamental aspects of lung cancer. Because it has been written by authors working very actively in the field it is remarkably up to date and provides excellent reviews of some topics of current major interest.

The first three chapters discuss various aspects of the biology of lung cancer by members of the National Cancer Institute group studying this problem. The first one is an excellent summary of their very successful methods of growing and characterising small cell lung cancer. They discuss a variety of techniques including a serum-free medium they have developed for growing small cell lines and the important problem of autocrine stimulation with the various growth factors that are produced. The second chapter also from the same group is a brief introduction to the possibilities of the use of monoclonal antibodies against lung cancer, whilst the third of the chapters, by Sherwin and Todaro, discusses the very important subject of transforming growth factors and their presence in human lung cancer cells and relation to growth.

A chapter on the tumour cloning assay from San Antonio discusses this very currently contentious topic in a somewhat optimistic way. However it does document the results obtained by that group and illustrates the potential use if reproducible techniques could be obtained. The group from Dartmouth is responsible for another chapter which discusses the role of the neurophysins in small cell lung cancer, these being yet another group of markers potentially of use.

Chapters on various aspects of therapy include those on the role of autologous bone marrow transplantation, the potential role of surgery in small cell lung cancer and a brief overall review of chemotherapy/radiotherapy in non-small cell lung cancer. Other chapters of use include an excellent one on the pathological diagnosis of poorly differentiated carcinoma of the lung.

This book is a pleasure to read. It is fairly up to date in an important subject which is now moving rapidly. It is very much recommended to both those working in the field and those who wish to find out what is happening in current investigations in this important subject.

N.M. Bleehen
University Department and
MRC Unit of Clinical Oncology
and Radiotherapeutics,
Addenbrooke's Hospital,
Cambridge.